# Nutritional quality indicators as complementary functional units for the sustainability assessment of grain-based foods in Sweden

**DOI:** 10.1016/j.crfs.2026.101310

**Published:** 2026-01-12

**Authors:** Martina Cassarino, Gianluca Giuberti, Margherita Dall’Asta, Hanieh Moshtaghian, Marta Bianchi

**Affiliations:** a Department of Sustainable Food Process, DISTAS, Università Cattolica del Sacro Cuore, Via Emilia Parmense, 84, Piacenza, 29122, Italy; b Department of Food Research and Innovation, RISE Research Institutes of Sweden, Frans Perssons väg 6, Gothenburg, Box 857, Borås, 501 15, Sweden; c Department of Animal Science, Food and Nutrition, DIANA, Università Cattolica del Sacro Cuore, Via Emilia Parmense, 84, Piacenza, 29122, Italy

**Keywords:** Nutritional quality, Indicators, Carbon footprint, Functional unit, Grain foods

## Abstract

Grain-based products, particularly whole grains, are integral to a healthy and sustainable diet. However, their nutritional quality and environmental impact vary, necessitating consideration of both aspects in sustainability assessments. This study aimed to identify nutritional indicators for grain-based foods and evaluate their suitability as functional units for carbon footprint analysis of Swedish products. Six indicators—CFQS-5, Keyhole, NRF11.3, NR-FI_carb_, Nutri-Score, and Nutri-Score_wholegrain_—were applied to 356 foods. Nutri-Score classified the highest proportion of products as high-quality (40 %), while Keyhole classified the fewest (13 %). Correlation with wholegrain content was strongest for CFQS-5 and weakest for Nutri-Score_wholegrain_. NRF11.3 and NR-FIcarb emerged as the most suitable functional units for carbon footprint calculations, showing moderate, statistically significant positive correlations with wholegrain content (ρ = 0.452 for NRF11.3, ρ = 0.475 for NR-FIcarb, both p < 0.001) and effectively rating grain-food categories in line with dietary guidelines. Transitioning from a weight-based to a nutrition-based functional unit, expressed as either NRF11.3 or NR-FIcarb, did not alter the finding that rice and sweet bakery products had the highest greenhouse gas emissions. However, it highlighted the favorable nutritional quality of “hard bread and crusts” and “flour, cereals, grains, and crushed cereals,” leading to reduced carbon footprints. These findings demonstrate that NRF11.3 and NR-FIcarb are equally effective in assessing the quality of grain-based foods and can be applied in food life cycle assessments, providing a practical tool for identifying foods with lower climate impact and higher nutritional value.

## Highlights

• Nutri-Score rated most grain products as high quality; Keyhole rated the fewest.
• Wholegrain content correlated most with CQFS-5 and least with Nutri-Score_wholegrain_.
• NRF11.3 and NR-FI_carb_ were most suitable to be applied as functional units to carbon footprints.
• Hard bread, flour, and porridge had lowest carbon footprint per unit of nutritional quality.
• Buns, pastry, and rice had highest carbon footprint per unit of nutritional quality.

## Graphical abstract

**Figure:**
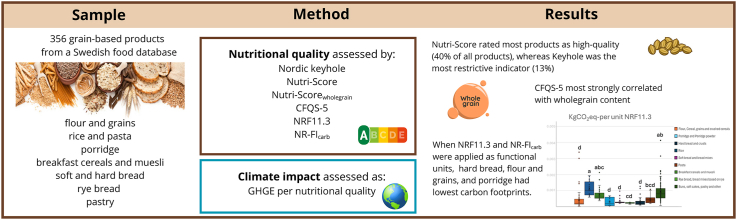

## 1 Introduction

Grain-based foods are a key component of sustainable healthy diets, providing carbohydrates, proteins, fibers and essential micronutrients (Poutanen et al., 2022). Wholegrain products, in particular, are rich in bioactive compounds, vitamins and minerals, such as vitamin E, some B vitamins, magnesium and zinc (McKevith, 2004; Dall’Asta et al., 2022), and they exert well-known health benefits beyond the role of fiber. Diets low in whole grains are responsible for one-fifth of the total burden of disease attributed to dietary factors globally, and are the greatest overall contributor to ischemic heart disease and colon and rectum cancers (Blomhoff et al., 2023; Hullings et al., 2020). According to data from the Global Burden of Disease Study, low whole grains consumption represents Sweden's leading risk factor to diet-related mortality (Institute for Health Metrics and Evaluation, 2021), highlighting whole grains promotion as critical in public health nutrition strategies for the Swedish population (Swedish Food Agency, 2025).

Most dietary guidelines recommend the consumption of wholegrain products, although a specific quantity is rarely reported (Ross et al., 2017). On the contrary, the Nordic Nutrition Recommendations (NNRs) suggest consuming at least 90 g/day (dry weight) of whole grains (including whole grains in products), quantity that is motivated by expected health outcomes and that may contribute to a healthy plant-based diet (Blomhoff et al., 2023). On the same line, the newly updated Planetary Health Diet increases the recommended daily consumption of whole grains to an average of 210 g/day (dry weight) (Rockström et al., 2025).

From an environmental perspective, grain-based foods have a relatively low carbon footprint. A 2015 study analyzed several categories of products using life cycle assessment (LCA), and identified grain-based products as supplying energy at a significantly lower carbon cost than other food categories (Drewnowski et al., 2015). Similarly, in a large-scale analysis of 57.000 food items commercialized in the UK and Ireland, authors found that cereals and bread had among the lowest greenhouse gas emissions per 100g, although with some variability in these product categories (Clark et al., 2022). As the interest of consumer, food industry and retail in products with low environmental impact that do not compromise safety and nutritional quality has progressively increased, the sustainability assessment of grain-based foods should be done holistically, including nutrition and health impacts (De Boer, 2003).

Nutritional density has therefore become, in recent years, a dimension that merges two areas of research: nutritional science and environmental science (Nemecek et al., 2016; Hallström et al., 2018). Consequently, one of the available methods for integrating these two areas is the choice of a nutritional functional unit. Nutritional quality indicators are widely used in nutritional LCA as functional units, both across the board or in a food group-specific approach (McLaren et al., 2021). Nutritional quality indicators often describe the nutritional adequacy of a food by estimating its nutrient contribution to the daily recommended intake of a specific population group. Frequently, indicators include both nutrients whose consumption is to be encouraged (i.e., with positive health effects) or to be limited (due to adverse health effects) (Cassarino et al., 2024).

Currently, there is no standardized method to integrate the nutritional quality of grain-based foods into LCA (Bianchi et al., 2020). On the other hand, in Sweden, front-of-package nutrition labelling systems (FOPNL) (e.g., Keyhole) have been used to assess the nutritional quality within this food category, but not in relation to environmental aspects (Pitt et al., 2023).

Therefore, the objective of this work was to identify indicators that describe the nutritional quality of grain-based foods and assess their relative performance in effectively capturing key aspects of nutritional quality (e.g., whole-grain content). The selection of indicators was intentionally broad, encompassing front-of-pack nutrition labelling (FOPNL) systems, grain-specific indicators commonly applied in nutrition research, and indicators with established use in food life cycle assessment (LCA). This approach enabled an analysis of the alignment between indicators suitable as functional units for Sweden-representative carbon footprints and nutritional quality indicators developed for other purposes (e.g., consumer communication or product improvement). The results provide insights into the substantial variability in nutritional quality and environmental performance among grain foods, offering guidance for selecting options that optimize both dimensions.

## 2 Literature review

In recent years numerous studies have used nutrient quality indicators as complementary functional units in food LCA (McLaren et al., 2021). One of the most widely used indicators for this purpose is the Nutrient Rich Food (NRF) index (Fulgoni et al., 2009). Different versions of NRF have been developed over the years to increase consistency with the dietary guidelines and/or tailor to specific product categories or target populations (Drewnowski et al., 2009). In 2020, Bianchi et al. (2020), assessed 45 NRF variants and presented NRF11.3 as the index most coherent with the Swedish dietary guidelines. Only a selection of grain-based foods was included in this study, which did not thoroughly assess the index performance in whole grains vs. refined grains. Alongside nutritional quality indicators for broad application, authors have suggested adopting a food group-specific approach in LCA, and presented an indicator for carbohydrate-rich foods (Kyttä et al., 2023b). The NR-FI_carb_ index includes nutrients for which cereals and potatoes are the main dietary sources in Finland. This nutrient selection is considered relevant for other Nordic countries as well, given that consumption patterns are broadly similar.

Despite the importance of whole grains, nutrient quality indicators do not commonly include whole grain content in their algorithms, probably due to differences in legislation regarding whole grains definitions and limited available composition data (Mathews and Chu, 2020). In indicators such as the NRF, other components are accounted for, including fiber, potassium, folate, added sugars, and sodium. Some attempts to integrate whole grains as part of a nutritional indicator have nevertheless been made. For example, the Carbohydrate Food Quality Score (CFQS-5) proposed by Drewnowski et al. (2022), included whole grains among five nutritional components relevant to grain-based foods.

Nutrient profiling (NP) labelling systems used as FOPNL may be better suited to capture nutritional quality aspects of grain-based products, although their application to food LCA is scarce (El-Abbadi et al., 2020). In the European context, Nutri-Score, a voluntary FOPNL adopted by eight countries, does not include whole grains in the current form, although its points scale for fiber has been optimized to allow a good discrimination between whole-grain and refined products (Scientific Committee of the Nutri-Score, 2021). Nevertheless, some authors have observed a persistent shortfall in Nutri-Score's ability to adequately grade the nutritional quality of products such as pasta, rice and flour and suggested further improvements to the points scale for carbohydrate foods (Amberntsson et al., 2025). In an earlier work by Kissock et al. (2022), an improved Nutri-Score was integrated to include whole-grain among the beneficial components of the algorithm. In a Nordic context, the Nordic Keyhole is a binary FOPNL that includes food group-specific thresholds for whole-grain content among the eligibility criteria, ensuring that only grain products with meaningful content of whole grains, and limited content of detrimental nutrients, can be labelled as healthy choices (Swedish Food Agency, 2018).

## 3 Methods

### 3.1 Selection of nutritional quality indicators

To identify potentially relevant studies presenting nutritional quality indicators applied to grain-based foods, a literature search was conducted informed by the PRISMA statement for scoping reviews (PRISMA-ScR) (McGowan et al., 2020). The databases Scopus, PubMed and SciFinder were searched for the period 2014–2024 using predefined keyword combinations. A total of 1320 articles were identified. After removal of duplicates (n = 568), 753 records were screened for title and abstract against specific inclusion and exclusion criteria, and 19 articles were retained for full-text analysis. The full selection process is shown in Supplementary Fig. S1. Detailed information on keywords, inclusion and exclusion criteria, and the characteristics of the 19 included studies is also given in Supplementary. These articles formed the basis for identifying nine nutritional indicators (Table 1). Finally, six indicators were selected based on the following criteria: if they are currently in use as FOPNL in either a Nordic or European country (Nordic Keyhole (Swedish Food Agency, 2018) and Nutri-Score (Santé publique France, 2025)); if they represent a novel indicator developed for grain-foods specifically (Nutri-Score_wholegrain_ (Kissock et al., 2022) and Carbohydrate Food Quality Score (CFQS – 5) (Drewnowski et al., 2022)); if they have been developed for and applied in nutritional LCA of grain-based foods (NRF 11.3 (Bianchi et al., 2020), NR-FI_carb_ (Kyttä et al., 2023b)). Three indicators did not qualify for these inclusion criteria or had other limitations as detailed in Table 1 and were therefore excluded.

**Table 1:** Nutritional quality indicators for grain-based foods identified from the literature, and the reason for inclusion or exclusion.

Nutrient quality indicator	Included in the study	Reason for inclusion/exclusion	Reference
Nordic Keyhole	YES	FOPNL used in Sweden. One of the key criteria for obtaining the Keyhole label is the wholegrain content.	Swedish Food Agency (2018)
Nutri-Score	YES	FOPNL used in several European countries. The latest algorithm has been improved to differentiate between wholegrain and refined products.	Santé publique France (2025)
Nutri-Score_wholegrain_	YES	Further variant of Nutri-Score where wholegrain content has been included in the beneficial component of the algorithm.	Kissock et al. (2022)
Carbohydrate Food Quality Score (CFQS)- 5	YES	Based on a simple calculation including wholegrain, potassium, and sodium alongside free sugars and fiber.	Drewnowski et al. (2022)
NRF11.3	YES	Recommended for use as functional unit in food LCA for a Swedish context.	Bianchi et al. (2020)
NR-FI_carb_	YES	Group-specific nutritional quality indicator recommended for food LCA of carbohydrate-rich foods	Kyttä et al. (2023b)
Health Star rating	NO	FOPNL applied to a geography that was not relevant to this study.	Curtain and Grafenauer (2019)
NutrInform Battery	NO	FOPNL based on a traffic light system, not a summary indicator.	Angelino et al. (2023)
SENSE algorithm	NO	Uses 20g as dietary reference for fiber, unlike many nutritional quality indicators.	Darmon et al. (2018)

### 3.2 Selection of grain-based food products

A total of 378 grain-based foods were obtained from the Swedish Food Composition Database (Swedish Food Agency, 2024). As nutritional quality indicators are often developed based on the nutrient needs of the adult population, 22 products targeted to children under three years of age were excluded. This resulted in 356 food items included in this study which were divided into 9 categories (Table S2), following the food categorization by the Keyhole regulation (Swedish Food Agency, 2018) with the addition of a category for buns, soft cakes, pastry and other, gathering products presently not covered by this Nordic FOPNL. The content of energy, macro- and micronutrients, added sugar and whole grains per 100 g was derived from the Swedish Food Composition Database (Swedish Food Agency, 2024). Composition data refer to both uncooked (n = 173) and cooked (n = 183) products, and no conversion to a prepared-to-eat form was made for products requiring cooking. Moreover, ingredients and recipes for composite foods and meals were obtained from the Swedish Food Agency (personal communication).

### 3.3 Assessment of the nutritional quality by six selected indicators

The nutritional quality of grain-based foods was evaluated using six selected indicators (Table 1). Algorithms and scales for point attribution are presented in Supplementary (Table S3). A short description of the indicators is provided below.

#### 3.3.1 Nordic Keyhole

The Nordic Keyhole label is a binary FOPNL aimed at promoting healthier food choices by facilitating the purchase of products with a better nutritional profile, in line with the Nordic Nutrition Recommendations (Blomhoff et al., 2023; Swedish Food Agency, 2018). The Keyhole does not apply to all foods, and among grain-based products, sweets and snacks are excluded (Wanselius et al., 2022) and were therefore not considered Keyhole eligible in this study. For the remaining grain-based food categories, eligibility for Keyhole was evaluated based on composition-related prerequisites (e.g., the percentage content of whole grains in the product) and group-specific nutrient criteria with established threshold values (e.g., the amount of saturated fat) (Table S3). The nutrient criteria vary among categories of grain-based products with a focus on fiber, fat, sugar and salt content.

#### 3.3.2 Nutri-Score

Nutri-Score is the most broadly used voluntary FOPNL in Europe (Chantal et al., 2017). It is a summary indicator resulting in a scale of 5 colors ranging from dark green to dark orange and associated letters ranging from A to E (Hercberg et al., 2022).

Nutri-Score is a measure of the overall nutritional quality of foods, calculated by taking into account the presence of unfavorable elements (energy, total sugars, saturated fatty acids, salt) and favorable elements (proteins, fibers, fruits, vegetables and oils derived from these ingredients) in 100g of product (Santé publique France, 2025).

In this study, the latest Nutri-Score algorithm was applied (Scientific Committee of the Nutri-Score, 2021). The content of fruits, vegetables, and legumes was calculated per 100 g product based on the Swedish Food Agency's ingredient lists and product recipes (see section 3.2).

#### 3.3.3 Nutri-Score_wholegrain_

Kissock et al. suggested a modification to the Nutri-Score algorithm by including whole grains as an additional component in the calculation (Kissock et al., 2022). In their study, up to −5 points were assigned to all foods containing 25–100 % whole grains on a dry weight basis using a non-linear sliding scale. In the present study, however, we modified the scale by assigning points to intermediate contents of whole grains. Specifically, two thresholds were added to the original scale, at 37.5 % and 75 % whole-grain content, corresponding to the attribution of −2 and −4 points respectively. The addition of intermediate thresholds enabled even smaller improvements in the formulation of whole-grain products.

#### 3.3.4 CFQS-5

The Carbohydrate Food Quality Score (CFQS-5) represents a novel indicator specifically developed for carbohydrate-rich foods with no known application in food LCA or as FOPNL (Drewnowski et al., 2022). This indicator was calculated based on the content of fiber, added sugars, sodium, potassium, and whole grains per 100 g product and attributed 1 point for each component according to specific thresholds and up to maximum 5 points. Added sugars replaced free sugars in the original algorithm as their content is available in the Swedish Food Composition Database and can be regarded as a more accurate indicator of product quality in grain-based foods.

#### 3.3.5 NRF11.3

NRF11.3 was calculated per 100 g of grain-based foods based on the content of 11 beneficial nutrients tailored to the Swedish population (Bianchi et al., 2020; Strid et al., 2021), and included protein, fiber, vitamins A, C, E, D, calcium, iron, magnesium, potassium and folate. Additionally, three nutrients to limit are accounted for in NRF11.3: saturated fat, added sugars and sodium. The beneficial nutrients' contribution to the dietary reference intake (DRI) and the nutrients to limit's contribution to the maximum recommended intake (MRI) were then calculated and their sums subtracted according to the algorithm presented in Supplementary (Table S3). DRIs and MRIs were obtained from the Nordic Nutrition Recommendations (Blomhoff et al., 2023) as average values between adult men and women (31–60 years old) and with a sedentary level of physical activity.

#### 3.3.6 NR-FI_carb_

NR-FI_carb_ was introduced as a nutritional quality indicator tailored to carbohydrates-rich foods and suitable for application in food LCA (Kyttä et al., 2023b). The index was calculated per 100 g of grain-based products as the arithmetic average of seven beneficial nutrients (carbohydrates, fiber, iron, magnesium, folate, phosphorus and potassium). In the original index, Kyttä et al. did not account for the nutrients to limit (Kyttä et al., 2023a); however, in subsequent work (Kyttä et al., 2023b), three nutrients to the list to limit (saturated fatty acids, sodium, and added sugar) were added. The same DRIs and MRIs applied for the calculation of NRF11.3 were also used here.

### 3.4 Criteria adopted to classify foods based on the selected indicators

The interpretation of optimal and sub-optimal nutritional quality in grain-based products differed depending on the indicator considered (Table 2). For the Keyhole label, the classification was binary, distinguishing between products that met criteria (YES) and those that did not (NO) (Pitt et al., 2023). For Nutri-Score and Nutri-Score_wholegrain_, products scoring A or B were classified as high nutritional quality, those scoring C, D or E were considered low quality, in alignment with the interpretation of the Nutri-Score (Chantal et al., 2017). For NRF11.3 and NR-FI_carb_ products were ranked and divided into quintiles (Q1 to Q5) from lowest to highest score. Products in Q4 and Q5 were considered as high nutritional quality, whereas products in Q1, Q2 and Q3 were considered as low nutritional quality (Bianchi et al., 2020). For CFQS-5, products achieving a score of 4 or 5 were deemed high quality, while those with scores 0,1, 2 or 3 were considered low quality (Drewnowski et al., 2022).

**Table 2:** Criteria for defining high vs. low nutritional quality according to the six selected indicators.

Indicator	High nutritional quality	Low nutritional quality
Nordic Keyhole	YES	NO
Nutri-Score	A - B	C - D -E
Nutri-Score_wholegrain_	A - B	C - D -E
NRF11.3	Q4 - Q5	Q1 - Q2 - Q3
NR-FI_carb_	Q4 - Q5	Q1 - Q2 - Q3
CFQS-5	4–5	0 - 1–2 - 3

### 3.5 Data collection on carbon footprints and integration with products’ nutritional quality

Carbon footprints for the 356 grain-based products were not available at the individual item level. Therefore, each of these was assigned 30 representative footprint values from the RISE Food Climate Database (RISE Research Institutes of Sweden, 2024). As a result, multiple items shared the same carbon footprint value as approximation. Climate impact was expressed as kilograms of carbon dioxide equivalent (CO_2_e) per kilogram of food product and included greenhouse gas emissions from primary production and up to the possible processing of raw food products in industry, excluding packaging and emissions from land use change. The climate data from the RISE Food Climate Database are intended to reflect Swedish consumption patterns and the dominant origin and production methods for foods available in Sweden. A climate contribution for a generally assumed transport to Sweden was included for imported food products. Only two of the selected indicators were suitable for use as complementary functional units, NRF11.3 and NR-FI_carb_, due to their numerical nature, where higher scores correspond to higher nutritional quality. An integrated climate impact per nutritional index unit was calculated by dividing kg CO_2_e per 100 g food product by NRF11.3 or NR-FI_carb_ per 100 g food product, following the same approach described by a previous study (Kyttä et al., 2023b). Higher nutritional quality scores implied relatively lower climate impacts per unit of nutritional index. To avoid negative values at the functional unit, nutritional quality scores for both indicators were adjusted so that the minimum value was set to 1.00. This implied adding 148.9 to NRF11.3 scores and 48.6 to NR-FI_carb_ scores.

### 3.6 Statistical analysis

Descriptive analyses (n, %) were performed to assess the proportions of products classified as high nutritional quality across and within groups, and the number of wholegrain food products for each selected indicator. Distributions of nutritional quality, climate impact, and water content were summarized as median and interquartile range (IQR) and compared across food groups. Data normality was assessed using the Kolmogorov–Smirnov test and, given the non-normal distribution, results were reported as median (interquartile range). Differences in Nutri-Score, Nutri-Score_wholegrain_, NRF11.3 and NR-FI_carb_ among groups of food groups (flour cereals, grains and crushed cereals; rice; breakfast cereals and muesli; porridge and porridge powder; soft bread and bread mixes; rye bread, bread mixes based in rye; pasta; buns, soft cakes, pastry, and other) were evaluated using the Kruskal–Wallis test with post-hoc pairwise comparisons using Bonferroni-adjusted significance tests, and two-group comparisons were performed using the Wilcoxon-Mann-Whitney *U* test.

Spearman correlation test was performed to explore the bivariate correlation between the five indicators (CFQS-5, NRF11.3, NR-FI_carb_, Nutri-Score and Nutri-Score_wholegrain_) and whole grains content within each product category. The relationship between the two variables (whole-grain content and nutritional quality indicators) was visualized by using scatter plot, including the linear regression line and the corresponding coefficient of determination (R^2^) to quantify the proportion of explained variance. The statistical analyses were conducted with IBM SPSS Statistics (v25.0; IBM Corp., Chicago, IL, USA) with significance set at p < 0.05.

## 4 Results and discussion

### 4.1 Products classified as high nutritional quality based on the selected indicators

Six nutritional quality indicators were identified from the literature. Two of these have been specifically developed for, and applied in, nutritional LCA of grain-based foods (NRF 11.3 (Bianchi et al., 2020), NR-FI_carb_ (Kyttä et al., 2023b)). The remaining four indicators include FOPNL used in a Nordic or European country (Nordic Keyhole (Swedish Food Agency, 2018) and Nutri-Score (Santé publique France, 2025)) as well as novel indicators developed specifically for grain-foods (Nutri-Score_wholegrain_ (Kissock et al., 2022) and Carbohydrate Food Quality Score (CFQS – 5) (Drewnowski et al., 2022)). Whereas Keyhole is a binary Yes/No label, and CFQS-5 is based on a simplified 0–5 scale, the remaining four indicators are summary indicators based on a continuous distribution of numeric scores.

A total of 356 foods were collected and classified into nine distinct food categories based on the grouping proposed by the Keyhole regulation, with the addition of the category “buns, soft cakes, pastry and other”. As a measure of the performance of the selected indicators, the percentage of products classified as high nutritional quality was evaluated across and within groups based on the ranking obtained for each indicator (Table 3). Across all food categories, Nutri-Score rated the highest number of high-quality products (39.8 %), whilst the rating according to the Keyhole showed the lowest number of high-quality products (12.6 %). Therefore, the Keyhole appears to be more restrictive than the other indicators. Similar results were observed in a larger sample of foods from various categories (Pitt et al., 2023) and in plant-based dairy alternatives (Moshtaghian et al., 2025). Both studies compared Keyhole and Nutri-Score and concluded that the former appears to be more restrictive, possibly due to the category-specific criteria for assessing product eligibility. It is worth noting that the rating of all products according to NRF11.3 and NR-FI_carb_ follows the predefined division in quintiles, where 40 % of products are included in quintiles 4 and 5 and defined as high nutritional quality.

**Table 3:** Products classified as high nutritional quality according to six indicators.

Indicator	All products (n = 356)	Flour, cereals, grains and crushed cereals (n = 62)	Rice (n = 28)	Breakfast cereals and muesli (n = 34)	Porridge and porridge powder (n = 23)	Soft bread and bread mixes (n = 35)	Rye bread, bread mixes based on rye (n = 8)	Hard bread and crusts (n = 41)	Pasta (n = 18)	Buns, soft cakes, pastry and other (n = 107)
CFQS-5^a^_, % (n)_	25.0 % (89)	56.4 % (35)	0 % (0)	50.0 % (17)	21.7 % (5)	11.4 % (4)	50.0 % (4)	48.7 % (20)	11.1 % (2)	1.8 % (2)
Keyhole^b^_, % (n)_	12.6 % (45)	33.8 % (21)	3.5 % (1)	20.5 % (7)	0 % (0)	2.8 % (1)	50.0 % (4)	24.3 % (10)	5.5 % (1)	0 % (0)
NRF11.3^c^_, % (n)_	39.8 % (142)	67.7 % (42)	17.8 % (5)	70.5 % (24)	8.7 % (2)	28.5 % (10)	62.5 % (5)	78.0 % (32)	27.7 % (5)	15.8 % (17)
NR-FI_carb_^d^_, % (n)_	39.8 % (142)	80.6 % (50)	50.0 % (14)	73.5 % (25)	8.7 % (2)	17.1 % (6)	50.0 % (4)	73.1 % (30)	44.4 % (8)	2.8 % (3)
Nutri-Score^e^_, % (n)_	39.8 % (142)	91.9 % (57)	64.2 % (18)	35.2 % (12)	30.4 % (7)	31.4 % (11)	62.5 % (5)	36.5 % (15)	88.8 % (16)	0.9 % (1)
Nutri-Score_wholegrain_^f^_, % (n)_	29.4 % (105)	82.2 % (51)	57.1 % (16)	11.7 % (4)	13.0 % (3)	25.7 % (9)	12.5 % (1)	12.2 % (5)	83.3 % (15)	0.9 % (1)

a CFQS-5 scores 4 and 5 are considered as high nutritional quality.

b Eligibility to bear Keyhole label is considered as high nutritional quality.

c Quintiles 4 and 5 in NRF11.3 values distribution are considered as high nutritional quality.

d Quintiles 4 and 5 in NR-FI_carb_ values distribution are considered as high nutritional quality.

e Nutri-score A and B are considered as high nutritional quality.

f Nutri-Score_wholegrain_ A and B are considered as high nutritional quality.

Among the assessed grain-based food categories, “flours, cereals, grains and crushed cereals” showed the highest percentage of high-quality foods (range 33.8–91.9 % depending on the indicator), whereas “buns, soft cakes, pastries and other products” showed the lowest percentage of high nutritional quality products (0–15.8 % depending on the indicator).

The performance of the analyzed indicators varied considerably across product categories. Highest observed percentages of high-quality products for CFQS-5 (56.4 %), NR-FI_carb_ (80.6 %) and Nutri-Score (91.9 %) were for products in the category “flour, cereals, grains and crushed cereals”. This finding aligns with Drewnowski et al. (2022), who reported that cereal-based products generally achieved higher nutrient profiling scores, thereby supporting the classification of this category as nutritionally favorable within the CFQS-5 system. Most Keyhole eligible products were in the category “rye bread and bread mixes based on rye” (50.0 %). NRF11.3 rated most products in the category “hard breads and crusts” as high quality (78.0 %), whereas, according to Nutri-Score_wholegrain_, most high-quality products were in the category “pasta” (83.3 %). The lowest observed percentage of high-quality products for CFQS-5 was observed in the category “rice” (0 % high-quality products). Keyhole rated the least products among “porridge and porridge powders” and “buns, soft cakes, pastries and other” (0 % for both categories) as high-nutritional quality. It is important to keep in mind that, according to the Keyhole criteria, sweet bakery products are classified as discretionary foods and therefore not eligible to carry the label (Wanselius et al., 2022). On the other hand, the relatively low number of porridge products classified as having high nutritional quality can be attributed to their higher water content. Similarly, lowest percentages of high-quality products for NRF11.3 were observed in “porridge and porridge powders” (8.7 %), whereas NR-FI_carb_ ranked “buns, soft cakes, pastries and other” lowest (2.8 % of high-quality products). Nutri-Score and Nutri-Score_wholegrain_ performed most selectively in “buns, soft cakes, pastries and other” with only 0.9 % of products rated as high-nutritional quality.

Two of the selected nutritional quality indicators are currently used as FOPNL in Europe (i.e., Nordic Keyhole and Nutri-Score). In this study, their performance in rating products as high nutritional quality diverged significantly in most grain-foods categories (Table 3). For example, Nutri-Score classified both pasta and rice much more favorably than Keyhole. Other authors have already reported on the Nutri-Score's poor performance in discriminating between whole-grain and refined flour, rice, and pasta products, and have suggested revisions to its algorithm to improve its consistency with the dietary recommendations (Amberntsson et al., 2025).

The two indicators with established applications as functional units (i.e., NR-FI_carbs_ and NRF 11.3) showed only marginal differences in identifying the grain-based categories with the highest and lowest numbers of high-nutritional-quality foods.

### 4.2 Nutritional quality according to NRF11.3, NR-FI_carb_ and two Nutri-Score indicators

**Fig. 1:**
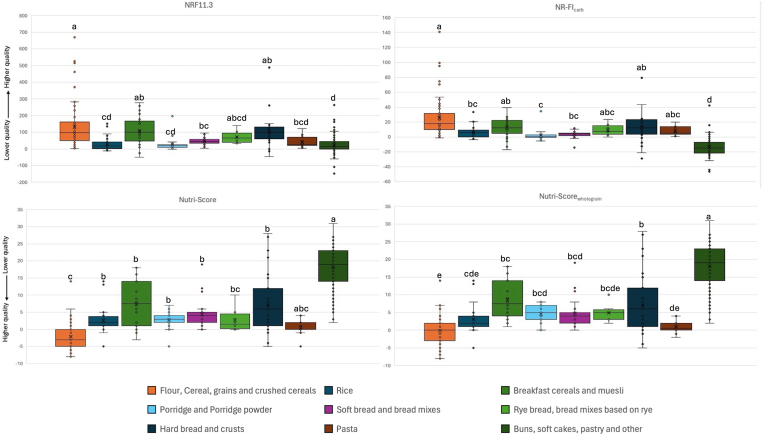
Nutrient quality in nine categories of grain-based foods assessed by four nutrient quality indicators. Distribution of scores for NRF11.3, NR-FI_carb_, Nutri-Score and Nutri-Score_wholegrain_ within food categories (“flour, cereal, grains and crushed cereals”, “rice”, “breakfast cereals and muesli”, “porridge and porridge powder”, “soft bread and bread mixes”, “rye bread, bread mixes based on rye”, “hard bread and crusts”, “pasta”, “buns, soft cakes, pastry and other”). For NRF11.3 and NR-FIcarb, higher values indicate better nutritional quality, whilst for Nutri-Score and Nutri-Scorewholegrain lower values describe a higher nutritional quality. Letters (a, b, c, d and e) indicate significate differences between all groups (∗p < 0.01).

Due to their continuous numerical nature, NRF11.3, NR-FI_carb_, Nutri-Score and Nutri-Score_wholegrain_ may better describe and finely capture differences in nutritional quality able to inform nutritional functional units in food LCA. Therefore, the distribution of scores for the four selected nutritional indicators was compared in the nine food categories (Fig. 1). For NRF11.3 and NR-FI_carb_, higher values indicate better nutritional quality, whilst for Nutri-Score and Nutri-Score_wholegrain_ lower values describe a higher nutritional quality. Median values of each selected indicator are reported in the Supplementary (Table S4).

According to NRF11.3 the category “breakfast cereals and muesli” presented the highest median (IQR) score [100.65 (48.40–167.01)], followed by “hard breads and crusts” and “flour, cereals, grains and crushed cereals” [99.44 (61.26–130.78), 97.55 (52.49–159.51)]. In contrast, “buns, soft cakes, pastry and other” presented the lowest median (IQR) value [14.13 (−0.85-43.23)]. Furthermore, NRF11.3 showed significant differences for the “buns, soft cakes, pastry and other” group compared to “hard bread and crusts” (p < 0.001), “breakfast cereals and muesli” (p < 0.001) and “flour, cereal, grains and crushed cereals” (p < 0.001), while no significant differences were found with the other categories (Fig. 1). These results align with prior work applying NRF11.3 within cereal subgroups. In a comparative analysis, Bianchi et al. (2020), reported that NRF11.3 ranked wholegrain breads, muesli and breakfast cereals as more nutrient-dense than refined cereals, whilst “sugar-containing products and snacks” were among the lowest-scoring items. This reference supports our interpretation that breakfast cereals and hard bread cluster at the top of the NRF11.3 distribution, whereas buns and soft cakes occupy the lower end.

Considering NR-FI_carb_, the median (IQR) value for “flour, cereals, grains and crushed cereals” [17.98 (10.29–31.50)] was highest, followed by “hard breads and crusts” and “breakfast, cereals and muesli” [12.27 (3.67–23.23), 12.08 (5.44–21.10)]. Similarly to NRF11.3, NR-FI_carb_ showed the lowest median (IQR) value for “buns, soft cakes, pastry and other” [−14.88 (−21.56 to −7.43)], which differed significantly from all other groups (p < 0.001) (Fig. 1). Furthermore, rye bread in our dataset ranked in the mid-to-high tier and did not differ significantly from most other categories. This aligns only partly with Kyttä et al. (2023b), who reported highest nutrient index scores for rye bread within the carbohydrate group.

On the other hand, according to Nutri-Score, the lowest median value (corresponding to highest nutritional quality) was observed in the category “flour, cereals, grains and crushed cereals” [−3.00 (−5.00-0)] and highest median value was in category “buns, soft cakes, pastry and other” [19.00 (14.00–23.00)]. For the latter category, statistically significant differences were found compared to all other categories (Fig. 1), except for “pasta”. This is likely due to the combination of unbalanced sample sizes (n_buns = 107 vs n_pasta = 18), which increases the standard error of the comparison and large variability in the “buns” group, which reduces the rank separation between the two distributions. According to Nutri-Score_wholegrain_, median (IQR) value [0 (−3.00-2.00)] for “flour, cereals, grains and crushed cereals” was lowest, whereas the highest value [19.00 (14.00–23.00)] was observed in the category “buns, soft cakes, pastry and other”, which was statistically different from all others. For both indicators, the median values reported above for the group “flour, cereal, grains, and crushed cereals” indicate that products in this category fall into the Nutri-Score A and B categories. This aligns with the study conducted by Øvrebø et al. (2023), in which flour and flour mixes scored A or B. In addition, in the “breakfast cereals and muesli” group we observed high within-category variability (IQR = 13 for Nutri-Score and IQR = 10 for Nutri-Score_wholegrain_), due to the different nutritional characteristics of the items included within each category. A similar result was reported by Angelino et al., who likewise documented high intra-category variability, confirming the marked heterogeneity of formulations (Angelino et al., 2023).

Overall, these results indicate a good alignment among the four numerical indicators in ranking grain-based product categories both with highest and lowest nutritional quality, and in synergy with dietary guidelines that emphasize reducing the consumption of sugary and high calorie cereal-based products.

### 4.3 Nutritional quality of food products in relation to wholegrain content

**Fig. 2:**
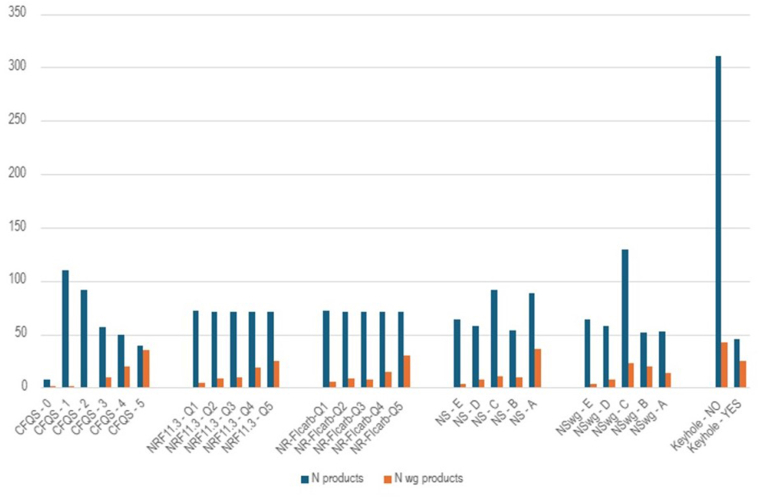
Distribution of wholegrain products across scoring for six nutrient quality indicators. Blue bars (N products) represent the total number of products per scoring level. Orange bars (N wg products) represent the number of wholegrain products (≥50 % whole grains per 100 on a dry weight basis) per scoring level. Scoring levels are ordered from low to high nutritional quality as follows: 0 to 5 for CFQS-5, E to A for Nutri-Score and Nutri-Score_wholegrain_, and Q1 to Q5 for NRF11.3 and NR-FI_carb_.

The five numerical indicators (i.e., all except the Keyhole) were evaluated for their ability to capture wholegrain content in food products. To this end, the number of wholegrain products identified by each selected indicator was analyzed (Fig. 2). Foods were defined as wholegrain if a wholegrain fraction of ≥50 % was present in the dry matter of the product.

Four of the analyzed indicators (CFQS-5, NRF11.3, NR-FIcarb, Nutri-Score) showed an increase in the number of wholegrain products in relation to improved nutritional quality. On the contrary, Nutri-Score_wholegrain_ showed the highest number of wholegrain products among those with intermediate nutritional quality (i.e., Nutri-Score C). Additionally, less than half of Keyhole-eligible foods were whole grains.

Correlation between wholegrain content and selected indices was performed (Supplementary Figs. S2–S6). Briefly, CFQS-5 showed the strongest positive correlation (p = 0.645, p < 0.001) whereas Nutri-Score_wholegrain_ showed the weakest and negative correlation (p = −0.128, p = 0.016).

Moderate and statistically significant positive correlations were found between the NR-FI_carb_ and whole-grain content (p = 0.475, p < 0.001), and a similar trend was observed for NRF11.3 (p = 0.452, p < 0.001). These findings suggest that both indices can capture well the nutritional quality of wholegrain products.

Nutri-Score, on the other hand, showed a moderate and statistically significant negative correlation (p = −0.340, p < 0.001), which is consistent with the fact that a lower score on this scale indicates higher nutritional quality, thus, products with more whole grains tend to score better.

Finally, the analysis of the Nutri-Score_wholegrain_ shows a very weak but statistically significant correlation with the content of whole grains (p = −0.128, p = 0.016), indicating that this index does not reflect the actual whole grain content in the analyzed products.

These results indicate that CFQS-5 exhibited the strongest correlation with the whole grains content, possibly explained by the narrow scale (0–5) of nutritional quality, which could have amplified the apparent strength of the correlation. However, being a simplified 0–5 scale, this indicator poorly discriminates between high and low nutritional quality and is therefore not well-suited for use as a functional unit. Conversely, NR-FI_carb_ and NRF11.3 represent the best options, both showing a moderate positive correlation with wholegrain content (Fulgoni et al., 2009). Finally, the Nutri-Score and Nutri-Score_wholegrain_ showed a negative correlation with wholegrain content, with the former presenting a stronger relationship than the latter. These results suggest that the Nutri-Score_wholegrain_ did not offer any additional advantage over the Nutri-Score in evaluating the nutritional quality of the analyzed grain-based foods, in line with the findings of Kissock et al. (2022), who reported that incorporating wholegrain content into the Nutri-Score algorithm produced minimal changes in overall product classification. Additionally, the reverse scale poses a challenge in the application of Nutri-Score as a functional unit. Indeed, products with higher nutritional quality (i.e. lower numerical values of Nutri-Score) would result in a relatively higher carbon footprint which conflicts with the purpose of applying nutrient-based functional units in nutritional-LCA.

### 4.4 Nutritional quality in relation to wholegrain in grain-based food categories

**Fig. 3:**
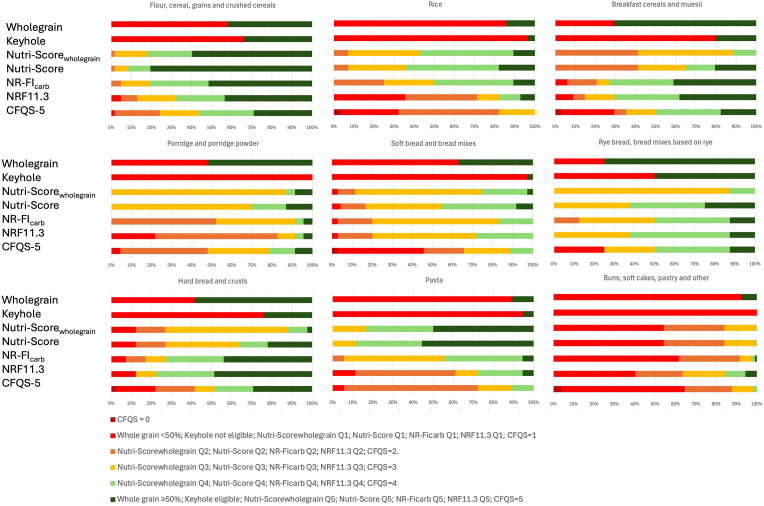
Comparison between whole grains and nutrient quality indicators in grain-based food groups. The classification of grain-based foods was calculated based on wholegrain content, Keyhole label eligibility, food distribution across CFQS-5, Nutri-Score, Nutri-Score_wholegrain_, and NRF and NR-FI_carb_ quintiles. Bars represent the percentage of food products per category of nutrient quality, with a scale ranging from dark red (lowest rating, with CFQS-5 = 0) to dark green (highest rating, with wholegrain ≥50 %; Keyhole eligible; Nutri-Score_wholegrain_ Q5; Nutri-Score Q5; NR-FI_carb_ Q5; NRF11.3 Q5; CFQS-5 = 5).

Fig. 3 shows the classification of grain-based food categories according to the content of whole grains, the eligibility to Keyhole, the foods distribution in levels of CFQS-5, Nutri-Score, Nutri-Score_wholegrain_, and in quintiles of NRF and NR-FI_carb_. In this analysis, products were classified as wholegrain based on the 50 % threshold, whereas the median content of wholegrain per category is provided in Supplementary (Table S4). As a result, the percentages reported in Fig. 3 and Table S4 differ, partly due to the different approaches and partly to the varying sample size across categories.

Importantly, NRF11.3 and NR-FI_carb_ were used in this study as relative measures of quality among grain products only, as the products stratification in quintiles was based on the included 356 food products. On the contrary, Nutri-Score classified foods as high or low nutritional quality based on thresholds developed across all foods and validated against the dietary guidelines, offering a basis for comparison beyond just grain-based products. For similarity to the Nutri-Score classification, a color scale from dark red (available only for CFQS-5) to dark green was applied to the other nutritional quality indicators.

“Rye breads and bread mixes based on rye” represented the category with the highest share of wholegrain products (75 %), the most Keyhole-eligible products (50 %), and an overall positive rating according to the other indicators (Fig. 3). A similar pattern was observed for “breakfast cereals and muesli”, “hard breads and crusts”, and “porridge and muesli”. On the other hand, “buns, soft cakes, pastry and other” contained the least wholegrain products (7.5 %), and coherently, no Keyhole eligible product and inferior scores according to the other indicators. The results for bread and sweet bakery products align with those of Dall’Asta et al. (Dall’Asta et al., 2022), whereas different results were found for the “breakfast cereals and muesli” category, probably due to the presence of different products within the category. Furthermore, many products belonging to the “buns, soft cakes, pastry and other” obtained a Nutri-Score E, which is consistent with the findings of Hafner and Pravst (2024).

“Flour, cereals, grains and crushed cereals” was rated positively by most nutritional indicators, even though less than half of the products in this category (41.9 %) were whole grains (Fig. 3).

Other food categories showed a higher discrepancy between the number of wholegrain products and the performance of the nutrient indicators. “Rice” and “pasta” contained a few wholegrain (14.3 and 11.1 % respectively) and Keyhole eligible products (3.6 and 5.6 % respectively) (Fig. 3). However, products in these categories showed a positive rating according to Nutri-Score and Nutri-Score_wholegrain_. Similar findings were demonstrated by Øvrebø et al. (2023), who analyzed the nutritional quality of foods using the latest Nutri-Score algorithm. In their study, the category “grains, pasta, rice and noodles” was classified with the Nutri-Score A or B. These results suggest that Nutri-Score may not adequately rank the nutritional quality of foods in categories that are widely consumed, such as pasta and rice, particularly in terms of wholegrain content.

### 4.5 Carbon footprint in relation to the nutritional quality

**Fig. 4:**
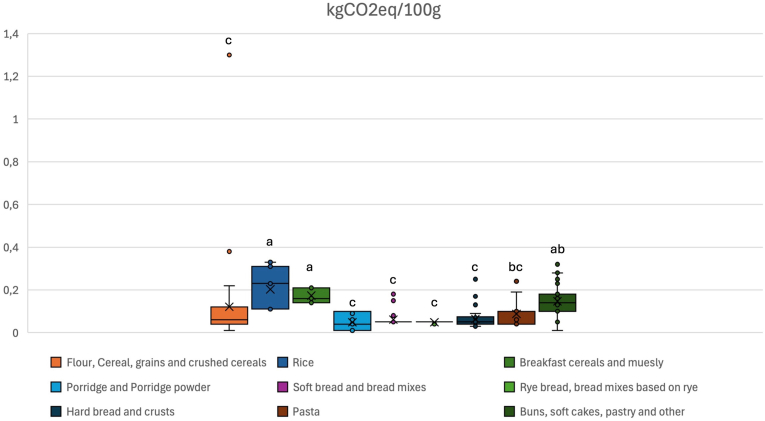
Carbon footprint of grain-based foods across product groups per 100g (kgCO2eq/100g). Climate impact per 100g (kgCO2eq/100g) of the nine food categories (“flour, cereal, grains and crushed cereals”, “rice”, “breakfast cereals and muesli”, “porridge and porridge powder”, “soft bread and bread mixes”, “rye bread, bread mixes based on rye”, “hard bread and crusts”, “pasta”, “buns, soft cakes, pastry and other”). The higher the median value, the greater the environmental impact of the category. Letters (a, b and c) indicate significate differences between all groups (∗p < 0.01).

**Fig. 5:**
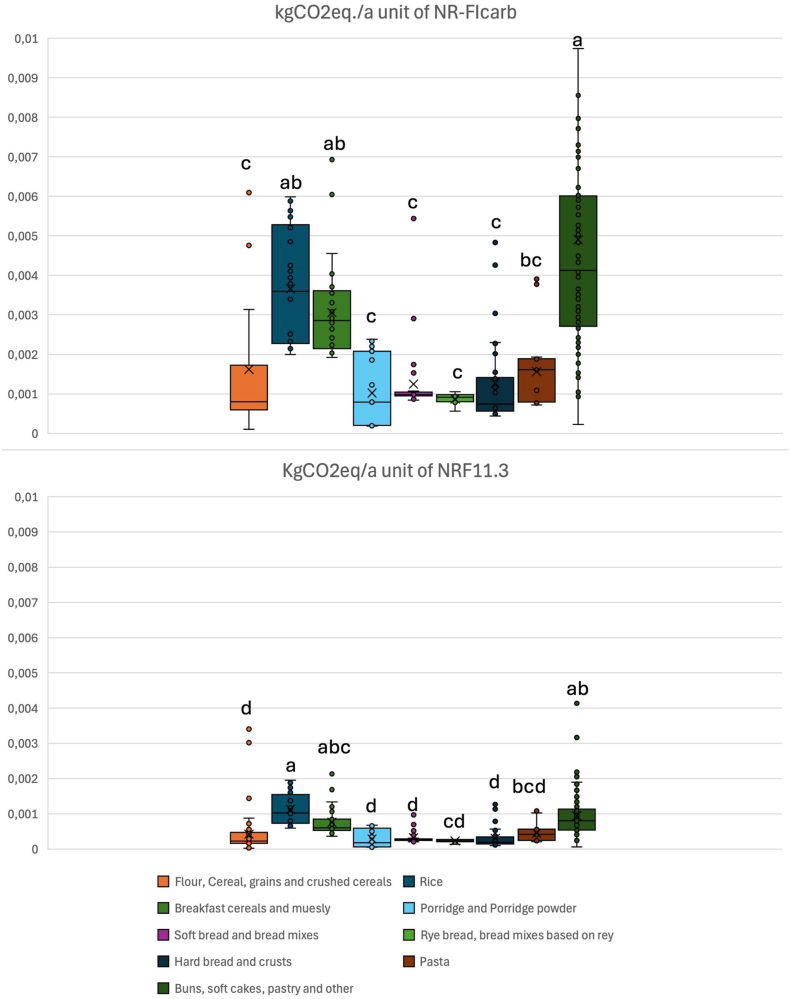
Carbon footprint of grain-based foods across product groups per unit of nutritional quality assessed by NR-FI_carb_ (kgCO2eq/NR-FI_carb_) and NRF11.3 (kgCO2eq/NRF11.3). Climate impact per NR-FIcarb (kgCO2eq/NR-FIcarb) and NRF11.3 (kgCO2eq/NRF11.) of the nine food categories (“flour, cereal, grains and crushed cereals”, “rice”, “breakfast cereals and muesli”, “porridge and porridge powder”, “soft bread and bread mixes”, “rye bread, bread mixes based on rye”, “hard bread and crusts”, “pasta”, “buns, soft cakes, pastry and other”). The higher the median value, the greater the environmental impact of the category. Letters (a, b, c and d) indicate significate differences between all groups (∗p < 0.01).

The climate impact of grain-based foods (expressed as kgCO_2_eq) was expressed for both 100g (Fig. 4) and for different nutritional functional units (Fig. 5). Based on the results presented in sections 4.1-4.4, NRF 11.3 and NR-FI_carb_ were the indicators selected for this purpose. The two Nutri-Score indicators were not applied as functional units, not only for the limitations discussed in paragraph 4.4, but also for the reverse classification scale (i.e., products with higher nutritional quality have lower scores). Products with a high nutritional quality would therefore result in relatively higher climate impacts if these were expressed per unit of Nutri-Score.

Median greenhouse gas emissions per kg product are reported in Supplementary (Table S10).

When expressed as kgCO_2_eq per 100g (Fig. 4), the “porridge and porridge powder” [0.04 (0.01–0.10)] and “rye bread” [0.05 (0.04–0.07)] categories presented the lowest median (IQR). On the other hand, the “rice” category presented the highest median (IQR) value [0.23 (0.11–0.31)], followed by “breakfast cereals and muesli” [0.16 (0.14–0.21)] and “buns, soft cakes, pastry and other” [0.14 (0.10–0.18)]. These findings align with a previous study by Kyttä et al. (2023b), which expressed impacts in kgCO_2_eq/100g of carbohydrate foods and found that rye bread had one of the lowest impacts, whereas rice ranked among the highest. To further investigate differences in kgCO2eq across food categories, pairwise comparisons were conducted using Bonferroni-adjusted significance tests. The analysis revealed several statistically significant differences (p < 0.05), indicating that some groups exhibit significantly distinct carbon footprints (Fig. 4). Interestingly, kgCO_2_eq/100g for “rice” did not differ significantly from “buns, soft cakes, pastry and other” (p = 0.45).

When expressed as kgCO_2_eq per unit of NR-FI_carb_ (Fig. 5), “hard bread and crusts” showed the lowest median (IQR) climate impact per unit [0.00074 (0.0006–0.0014)], followed by “flour, cereal, grains and crushed cereals” and “porridge and porridge powder” [0.000796 (0.0006–0.0017), 0.000794 (0.0002–0.0020)]. In contrast, the highest median climate impacts per unit were observed for “buns, soft cakes, pastry and other” [0.004 (0.0028–0.0060)] and “rice” [0.0035 (0.0023–0.0052)]. This pattern aligns with a previous study (Kyttä et al., 2023b), where authors assessed climate impacts per nutritional functional unit and similarly found that bread had the lowest and rice has the highest carbon footprint among cereal products. Unlike Kyttä et al. (2023b), however, in our study, rye bread did not show the lowest kgCO_2_eq per unit of NR-FI_carb_.

When expressed as kgCO_2_eq per unit of NR11.3 (Fig. 5), “porridge and porridge powder” had the lowest median (IQR) climate impact per unit [0.00018 (0.00006–0.0006)], followed by “hard bread and crusts” and “flour, cereal, grains and crushed cereals” [0.00019 (0.0001–0.0003), 0.00022 (0.0002–0.0004)]. In contrast, “rice” showed the highest climate impact [0.00102 (0.0007–0.0014)] followed by “buns, soft cakes, pastry and other” [0.00080 (0.0005–0.0011)].

Statistically significant differences in carbon footprints were observed across categories when NR-FI_carb_ and NRF11.3 were used as functional units. These differences followed a pattern similar to that observed for carbon footprints per 100g, with “rice” and “breakfast cereals and muesli” not differing significantly from “buns, soft cakes, pastry and other”. Notably, the carbon footprint of pasta was significantly lower than that of “buns, soft cakes, pastry and other” when expressed per unit of NR-FI_carb_ but not when expressed per 100g or unit of NRF11.3. Moreover, pasta showed lower median greenhouse gas emissions than rice, with this difference reaching statistical significance when expressed per 100 g and NRF11.3 (p < 0.05). This finding can be explained by the lower emissions associated with wheat cultivation compared with rice production in paddy fields (Clark et al., 2019; Hess et al., 2016).

Divergence in environmental performance across grain-based foods is nevertheless small compared with other product groups and categories and can be largely explained by differences in nutritional quality, which in turn arise from variations in ingredients and production processes. These findings align with Clark et al. (2022), who reported that cereal-based products generally perform better nutritionally and environmentally than highly processed food categories.

Grain-based foods with a limited number of ingredients and simple production processes, such as flour and grains, tend to have low greenhouse emissions (Poore and Nemecek, 2018). On the contrary, grain-based products containing high-impact ingredients such as added sugars, vegetable oils, and dairy (e.g., bakery products) tend to have a higher impact.

Switching from a weight-based to a nutritional functional unit did not alter the finding that “rice” and “buns, soft cakes, pastry and other” exhibited the highest greenhouse gas emissions. However, it highlighted the favorable nutritional quality of categories such as “hard bread and crusts” and “flour, cereal, grains and crushed cereals”, resulting in lower carbon footprints.

## 5 Limitations

One limitation of this study is that the grain-based products varied significantly in water content (Supplementary, Table S11). Both uncooked and cooked foods were included. Some categories largely consisted of raw products that do not require preparation before consumption (e.g. breakfast cereals and muesli), while others included mainly cooked products (e.g., porridge and porridge powders). The high scores of nutritional qualities observed in the category “flour, cereals, grains and crushed cereals” are certainly impacted by the low water content of flours and grains.

A further approximation in this study relates to the limited availability of carbon footprint data for the foods assessed. Only 30 distinct values of kg CO_2_-eq were assigned to the 356 products included in the analysis. This limited variability may have reduced the differentiation among grain-based products in terms of greenhouse gas emissions, implying that—when nutritional functional units were applied—differences in nutritional quality largely drove the observed variation among products. However, it is important to note that the range of carbon footprints for grain-based foods is relatively narrow compared with other product categories (0.01–0.31 kg CO_2_-eq per 100 g in this study), which also constrains the influence of approximated carbon footprint values on the results.

## 6 Conclusions

This study provides a comprehensive analysis of the relative performance of six nutritional quality indicators applied to nine categories of grain-based foods consumed in Sweden. Two of the indicators included (i.e., NRF 11.3 and NR-FI_carb_) have previously been applied in nutritional LCA of grain-based foods and are compared here with indicators developed for other purposes. These include front-of-pack nutrition labelling systems currently in use or under consideration for consumer guidance (i.e., the Nordic Keyhole and Nutri-Score), as well as novel indicators specifically developed for carbohydrate-rich foods (i.e., CFQS-5 and Nutri-Score_wholegrain_).

Overall, the results indicate a generally good level of agreement among the assessed indicators in identifying product categories with both high (e.g., “flours, cereals, grains, and crushed cereals”) and low (e.g., “buns, soft cakes, pastries, and other products”) nutritional quality, consistent with dietary recommendations on wholegrain consumption.

However, the Nordic Keyhole proved to be the most restrictive indicator, with only a small proportion of the assessed products qualifying for this front-of-pack nutrition label. Nutri-Score, by contrast, classified the largest number of products as high nutritional quality, including relatively generous ratings for pasta and rice—categories with a high share of refined products.

A further limitation of both Nutri-Score and Nutri-Score_wholegrain_ is their reversed numerical scales, which would require mathematical transformation for use as functional units. This approach was not pursued in the present study, as neither indicator appeared to offer clear advantages over NRF 11.3 or NR-FIcarbs, and such transformations would further limit the applicability of these indicators as functional units. In addition, Nutri-Score_wholegrain_ exhibited the weakest correlation with wholegrain content among the indicators assessed.

Among the five numerical indicators assessed (i.e., all except the Keyhole), CFQS-5 showed the strongest positive correlation with wholegrain content. However, its simplified five-point scale limits both its discriminatory power and its suitability for application as a functional unit in food LCA.

The results therefore indicate that NRF 11.3 and NR-FI_carb_ represent the most suitable nutritional functional units for application in food LCA of grain-based products. Both indicators exhibited moderate, statistically significant positive correlations with wholegrain content and were equally effective in ranking grain-food categories according to nutritional quality, in line with dietary guidelines (i.e., promoting wholegrain intake and discouraging consumption of sugar- and fat-rich bakery products).

Switching from a weight-based to a nutritional functional unit, whether expressed as NR-Fi_carb_ or NRF11.3, did not change the finding that “rice” and “buns, soft cakes, pastry and other” exhibited the highest greenhouse gas emissions. However, it emphasized the favorable nutritional quality of categories such as “hard bread and crusts” and “flour, cereal, grains and crushed cereals”, leading to reduced carbon footprints.

This work explores the integration of nutritional quality into the assessment of environmental impacts of grain-based products consumed in Sweden. With the overarching goal of promoting healthy and sustainable diets (Sustainable healthy diets: guiding principles, 2019), it is important to identify grain-based products that offer an optimal balance between nutritional quality and environmental sustainability. In this study, NRF11.3 and NR-FI_carb_ were used as operational metrics to compute n-LCA, therefore supporting product reformulation, and providing guidance toward more sustainable food consumption without neglecting nutritional and health aspects. Future studies should extend this methodology to other food categories, thereby contributing to the development of standardized criteria for incorporating the nutritional dimension into LCA.

## CRediT author statement

Martina Cassarino: Formal analysis, investigation, data curation, writing – original draft.

Gianluca Giuberti: Conceptualization, Writing – Review & Editing.

Margherita Dall’Asta: Validation, formal analysis, Writing – Review & Editing.

Hanieh Moshtaghian: Methodology, Writing – Review & Editing.

Marta Bianchi: Conceptualization, Methodology, Supervision, Writing – Review & Editing.

## Declaration of competing interest

The authors declare that they have no known competing financial interests or personal relationships that could have appeared to influence the work reported in this paper.

## Figures and Tables

**Figure:**
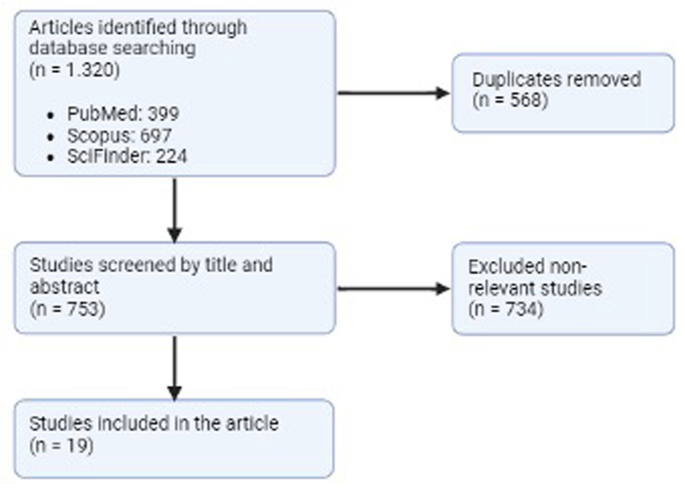
Figure S1. Flow chart of the literature search and selection process.

**Figure:**
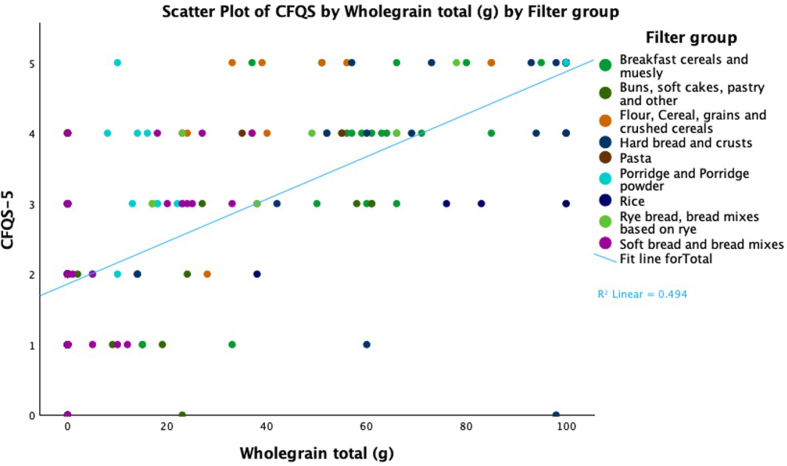
Figure S2. Relationship between total wholegrain content (g) and CFQS-5 score in different groups of cereal-based products. Scatter plot of cereal-based products according to their total wholegrain content and CFQS-5. Each point represents a food item; colors distinguish different food groups (breakfast cereals and muesli; buns, soft cakes, pastry and other; flour, cereal, grains, and crushed cereals; hard bread and crusts; pasta, porridge and porridge powder; rice; rye bread, bread mixes based on rye; soft bread and bread mixes). CFQS-5 shows the strongest positive correlation (p = 0.645, p < 0.001).

**Figure:**
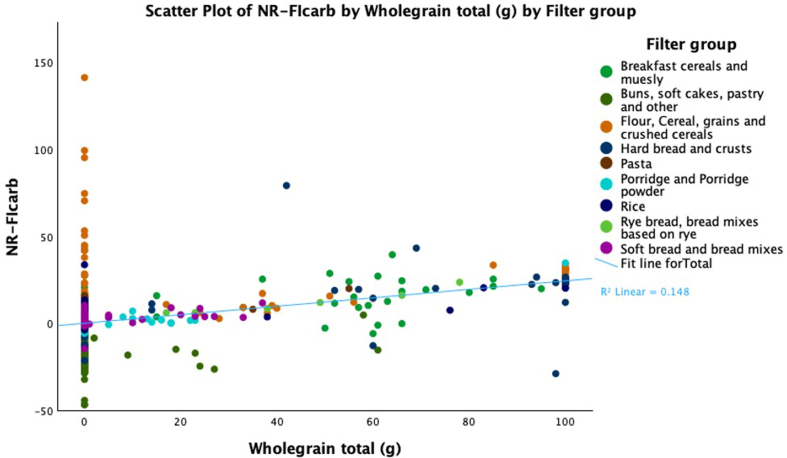
Figure S3. Relationship between total wholegrain content (g) and NR-FI_carb_ score in different groups of cereal-based products. Scatter plot of cereal-based products according to their total wholegrain content and NR-FI_carb_. Each point represents a food item; colors distinguish different food groups (breakfast cereals and muesli; buns, soft cakes, pastry and other; flour, cereal, grains, and crushed cereals; hard bread and crusts; pasta, porridge and porridge powder; rice; rye bread, bread mixes based on rye; soft bread and bread mixes). NR-FIcarb shows a positive correlation (p = 0.475, p < 0.001).

**Figure:**
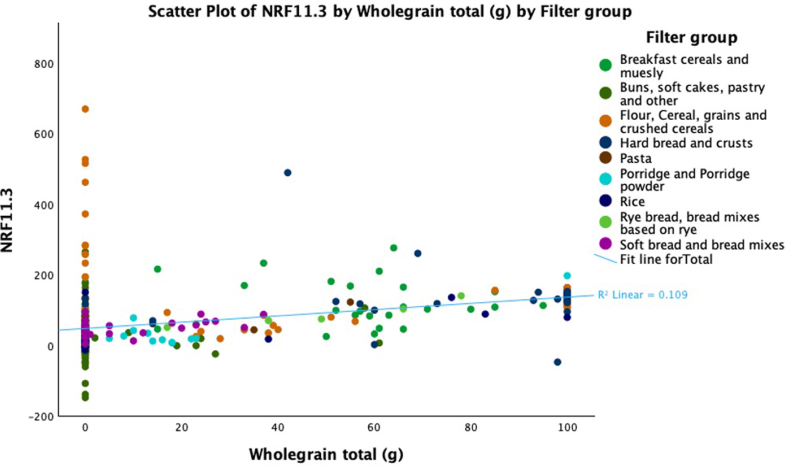
Figure S4. Relationship between total wholegrain content (g) and NRF11.3 score in different groups of cereal-based products. Scatter plot of cereal-based products according to their total wholegrain content and NRF11.3. Each point represents a food item; colors distinguish different food groups (breakfast cereals and muesli; buns, soft cakes, pastry and other; flour, cereal, grains, and crushed cereals; hard bread and crusts; pasta, porridge and porridge powder; rice; rye bread, bread mixes based on rye; soft bread and bread mixes). NRF11.3 shows a positive correlation (p = 0.452, p < 0.001).

**Figure:**
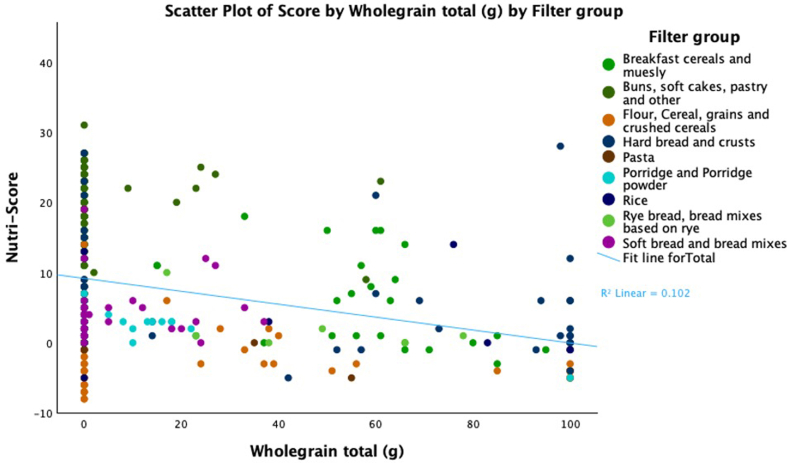
Figure S5. Relationship between total wholegrain content (g) and Nutri-Score score in different groups of cereal-based products. Scatter plot of cereal-based products according to their total wholegrain content and Nutri-Score. Each point represents a food item; colors distinguish different food groups (breakfast cereals and muesli; buns, soft cakes, pastry and other; flour, cereal, grains, and crushed cereals; hard bread and crusts; pasta, porridge and porridge powder; rice; rye bread, bread mixes based on rye; soft bread and bread mixes). Nutri-Score shows a negative correlation (p = −0.340, p < 0.001).

**Figure:**
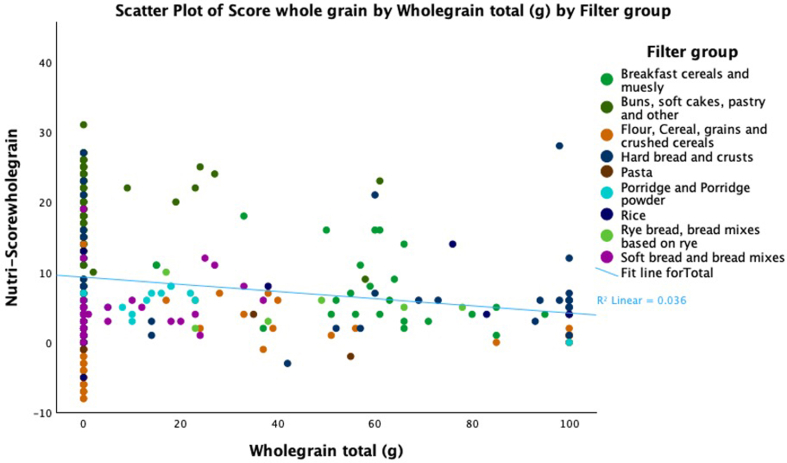
Figure S6. Relationship between total wholegrain content (g) and Nutri-Scorewholegrain score in different groups of cereal-based products. Scatter plot of cereal-based products according to their total wholegrain content and Nutri-Score_wholegrain_. Each point represents a food item; colors distinguish different food groups (breakfast cereals and muesli; buns, soft cakes, pastry and other; flour, cereal, grains, and crushed cereals; hard bread and crusts; pasta, porridge and porridge powder; rice; rye bread, bread mixes based on rye; soft bread and bread mixes). Nutri-Score_wholegrain_ shows a very weak correlation (p = −0.128, p = 0.016).
