# Supplementary material for: Nutritional quality indicators as complementary functional units for the sustainability assessment of grain-based foods in Sweden
Source: Curr Res Food Sci. 2026 Jan 12;12:101310. doi: 10.1016/j.crfs.2026.101310 (PMC12856264; doi:10.1016/j.crfs.2026.101310)
Supplement: Multimedia component 1 [file mmc1.docx]

Supplementary

## Literature search for the identification of nutritional quality indicators

The literature search was conducted through Scopus, PubMed and SciFinder and considered articles published between January 2014 and November 2024. The research was performed in two stages. Initially, the following keywords were used: “grain”, “nutrient profiling methods”, “nutrient indicators”, “carbohydrate foods”, “nutrient density”, “nutrient system”, separated by the Booleon operator “AND”. Subsequently, the research was extended by using the following keywords: “Nutri-score”, “Keyhole”, “NRF”, “label”, “LCA”, “nLCA”, “nFU”, separated also by the Booleon operator “AND”.

Articles were considered if published in peer-reviewed scientific journals and written in English. The two searches combined retrieved a total of 1320 articles. After screening for duplicates, 753 articles were retained. Titles and abstracts were assessed for inclusion and exclusion criteria. Articles were included if they reported the use of one or more nutritional indicators used to assess the nutritional quality of grain products. For grain products it was intended carbohydrate-rich foods excluding potatoes and including whole grains and cereal products. The exclusion criteria were as follows: (i) studies exploring the correlation between the consumption of carbohydrate-rich foods and health outcomes; (ii) studies on the quality carbohydrate-rich foods, assessed without the use of nutritional indicators; (iii) papers developing nutritional quality indicators based on a specific population; (iv) papers on LCA studies in non-grain products. After reviewing titles and abstract, 19 articles were retained and assessed in full text (Table S1 and figure S1).

**Figure S1 here.**

**Table S1**. Characteristics of the 19 included articles.

| **Author** | **Nutritional quality**  **indicator** | **Method** | **Food groups** |
| --- | --- | --- | --- |
| [1] | Nutri-Score and NutrInform battery | A total of 349 items were found; the online search for the information was performed in July 2022. The Nutri-Score was calculated for each item in accordance with the rules reported in the document. Data for NutrInform battery were calculated by considering the standard serving size or 30g as defined in the manual. | Breakfast Cereals |
| [2] | NRF | Evaluated nutrient density indexes by systematically assessing the role of methodological variables with the purpose of identifying the index able to rank foods with the highest coherence with the Swedish dietary guidelines. | Food groups |
| [3] | Health Star Rating | Assigned whole grains points and a favorable component of the HSR based on the whole-grain content of foods. Compared the original, and three modified HSR algorithms using independent-samples median test. | Whole grain |
| [4] | Carbohydrate Food Quality Indicators | A series of expert panel meetings and a scoping review of the literature focused on carbohydrate quality indicators and metrics produced in the last 10 years. | Carbohydrate Food |
| [5] | Carbohydrate Food Quality Score (CFQS) | This scoring system recognizes the value by using a four-point scale (i.e., CFQS-4) based on four nutrients of concern to assess the quality of non-grain CFs and a five-point scale (i.e., CFQS-5) based on the same nutrients of concern along with whole grain content for assessing the quality of grains foods. | Carbohydrate Foods |
| [6] | Health Star Rating | Products were collected between September 2017 – September 2018. Comparison of whole grain and refined grains foods from each subcategory displaying an HSR. | Bread, rice, pasta, noodles, flour, and breakfast cereals |
| [7] | SENS algorithm | The SENSE algorithm allocates a class to food according to the value of qualifying score, the SAIN_SENSE_, and a disqualifying score, LIM_SENSE_. | Beverages, added fats and other solid foods |
| [8] | Nutri-Score, Health Star Rating, Nutri Rich Food Index | How to integrate whole grains in NP models | Whole grains |
| [9] | Carbohydrate Food Quality Score (CFQS) | Two CFQS models: CFQS-4 (fiber, free sugars, sodium, potassium) and CFQS-5 (fiber, free sugars, potassium and whole grain), are applied to 2596 carbohydrate foods. | Cooked grain, bread and rolls, quick breads, ready-to-eat (RTE) cereals, cooked cereals, savory snacks, crackers, snack/meal bars, sweet bakery products, candy and other dessert. |
| [10] | 5-colour-nutrition label (5-CNL) | Nutritional information was collected through the internet and supermarket research for N=433 breakfast cereals. The FSA score and 5-CNL category allocation were computed for each cereal. Nutrient content and FSA score were compared across types of cereals. | Breakfast Cereals |
| [11] | NR-FI_prot_ and NR-FI_carb_ | To validate intake-based product-group-specific nutrient indices previously developed for protein (NR-FI_prot_) and carbohydrate (NR-FI_carb_) foods and for fruits and vegetables (NR-FI_veg_), they applied principal component analysis to investigate correlations between nutrients in foods and dishes representing a typical Finnish diet. | Proteins, carbohydrates and vegetables/fruits. |
| [12] | Nutri-Score | The whole grain content of food was added to the algorithm. Compared the original and the modified Nutri-Score to foods composition and dietary intake data from Austria, France, United Kingdom and United States. | Whole grain |
| [13] | NR-FI_carb_, NR-FI_veg_, NR-FI_prot_ | Develop product-group-specific indices to be used as the nFU in the LCA for product groups of vegetables, fruit and berries, and sources of carbohydrates. The practical application of these indices was then tested through demonstrative LCAs for the selection of different foods. | Vegetables, fruit and berries, and sources of carbohydrates. |
| [14] | Nutri-Score update | The update Nutri-Score was calculated for 1,872 foods in a Norwegian foods database. The discriminatory ability of the update Nutri-Score was considered by exploring the distribution of Nutri-Score within categories of foods using box plots and frequency tables, and by disqualifying components that contributed most to the Nutri-Score class. | 13 food groups: fruit, vegetables, cereals, grains and similar, fish, meat, eggs, dairy products, sauces and/or salty snacks, composite foods and meals, fats and oils, nuts and seeds, plain water, fruit and vegetables-based beverages, dairy beverages and plant-based alternatives. |
| [15] | Keyhole and Nutri-Score | The Keyhole and the update Nutri-Score 2022 algorithm was applied to 984 foods and their nutrient compositions, obtained from the food database of the Swedish Food Agency. Agreements and disagreements were calculated as a percentage for all items and by the food group. | 12 food groups: vegetables, flour, grain, rice, bread and pasta, milk, cheese and related plant-based products, fats, oils and spreads, meat, plant-based products, ready meals, dressing and sauces, other |
| [16] | Nutri-Score (initial and modified version) | An analysis of nutritional qualities using three indicators: Nutri-Score (initial and modified version), WHO Europe nutrient profile model, and Nova. | Breakfast Cereals |
| [17] | NRF | Evaluation of fifteen NRF index scores against the Dutch Healthy Diet Index (DHD-index). | Danish food |
| [18] | Nutri-Score | The data was collected using photographs taken from the front-of-pack, ingredient list and nutrition information panel of breakfast cereals. The overall nutritional quality of the breakfast cereals was calculated using the Nutri-Score. | Breakfast Cereals |
| [19] | Keyhole | Dietary data were derived from Riksmaten Adolescents 2016-2017, a cross-selection school-based dietary survey in Sweden. A nationally representative sample of Swedish adolescents in school years 5, 8 and 11 was recruited, with mean ages 12, 15 and 18 years respectively. The survey was conducted by the Swedish Food Agency. | Dietary survey |

1. Food groups

The products were classified into nine food categories, following the Keyhole regulation [20] with the addition of the category buns, soft cakes, pastry and other.

**Table S2**. Nine categories of grain-based foods.

| **Group** | **Categories** |
| --- | --- |
| 4 | Flour, cereals, grains and crushed cereals |
| 5 | Rice |
| 6 | Breakfast cereals and muesli |
| 7 | Porridge and porridge powder |
| 8a | Soft bread and bread mixes |
| 8b | Rye bread, bread mixes and other rye-based products |
| 9 | Hard bread and crusts |
| 10 | Pasta |
| 11 | Buns, soft cakes, pastry and other |

## Nutritional quality indicators

**Table S3**. Algorithms and thresholds for points attribution for six selected nutrient quality indicators.

| **Nutritional indicator** | **Composition-related pre-requisites** | **Nutrient criteria** |
| --- | --- | --- |
| Nordic Keyhole | **Food group 4**: flour, cereals, grains and crushed cereal containing at least 100% whole grains calculated on the dry matter of the cereal part. | Fiber at least 6 g/100 g |
|  | **Food group 5**: rice containing 100% whole grains calculated on the dry matter of the product. | Fiber at least 3 g/100 g |
|  | **Food group 6**: breakfast cereals and muesli containing at least 55% whole grain calculated on the dry matter of the product.  Gluten-free breakfast cereals and muesli must contain at least 20% whole grains calculated on the dry matter of the product. | Fat not more than 8 g/100 g  Sugars not more than 13 g/100 g, of which added sugars not more than 9 g/100 g  Fiber at least 6 g/100 g  Salt not more than 1,0 g/100 g |
|  | **Food group 7**: porridge and porridge powder containing at least 55% whole grains on the dry matter of the product. | Fat not more than 4 g/100 g  Sugars not exceeding 5 g/100 g  Fiber at least 1 g/100 g  Salt not more than 0,3 g/100 g |
|  | **Food group 8a**: soft bread and bread mixes to which only liquid and possible yeast are to be added and which contain at least 30% whole grains calculated on the dry matter of the product.  Gluten-free breads and bread mixes must contain at least 10% whole grains calculated on the dry matter of the product. | Fat not more than 7 g/100g  Sugars not exceeding 5 g/100 g  Fiber at least 5 g/100 g  Salt not more than 1,0 g/100 g |
|  | **Food group 8b**: rye bread, bread mixes, and other rye-based products to which only liquid and possible yeast are to be added. The product must contain at least 35% whole grain calculated on the product`s dry matter. The cereal part must contain at least 30% rye. | Fat not more than 7 g/100 g  Sugars not exceeding 5 g/100 g  Fiber at least 6 g/100 g  Salt not more than 1,2 g/100 g |
|  | **Food group 9**: hard bread and crusts and ready-made mix for such products where only liquid and possibly yeast should be added. The product must contain at least 50% of the whole grain calculated on the product`s dry matter. | Fat not more than 7 g/100g  Sugars not exceeding 5 g/100 g  Fiber at least 6 g/100 g  Salt not more than 1,3 g/100 g |
|  | **Food group 10**: pasta (without filling). The product must contain at least 50% of the whole grain calculated on the product`s dry matter.  Gluten-free pasta (without filling) has no requirement for whole grains. | Fiber at least 6 g/100 g  Salt not more than 0,1 g/100 g |
| **Nutritional indicator** | **Calculation for points attribution** | **Nutrients included** |
| NRF11.3 | Index= (∑1−x (Qualitative nutrient/DRI)) − (∑1−y (Disqualitative nutrient/MRI)) | Qualitative nutrients: protein, dietary fiber, vitamin A, C, E, D, calcium, iron, magnesium, potassium, folate.  Disqualitative nutrients: saturated fat, added sugar, Na. |
| NR-FIcarb (modified) | *Index = (∑* $\frac{Nutrient i}{DRI i} X 100)$ */ number of nutrients in the index – LIM3*    $LIM3= \sum i=\left( 1-3 \frac{Nutrient j}{MRV} X 100 \right)/3$ | Nutrient i= carbohydrates, fiber, Fe, Mg, P, K, and folate.  Nutrient j= saturated fat, added sugar, Na. |
| Nutri-Score | If the N component is ≥11 points, the formula is:  Nutri-Score = N – (Points Fiber + Points FVL)  If the N component is <11points, the formula is:  Nutri-Score = N – P | Unfavorable elements (N): energy in KJ, sugars, salt, saturated fatty acids and non-nutritive sweeteners for beverages.  Favorable elements (P): proteins, fibers, fruits, vegetables and legumes. |
| Nutri-Score_wholegrains_ | If the N component is ≥11 points, the formula is:  Nutri-Score = N – (Points Fiber + Points FVLWG)  If the N component is <11points e, the formula is:  Nutri-Score = N – P  Wholegrain content below 25%: 0 points;  Wholegrain content of 25% or more: -1 points;  Wholegrain content between 25 and 37,5%: - 2 points;  Wholegrain content between 37,5 and 50% - 3 points;  Wholegrain content between 50% and 75% - 4 points;  Wholegrain content between 75% and 100% - 5 points; | Unfavorable elements (N): energy in KJ, sugars, salt, saturated fatty acids and non-nutritive sweeteners for beverages.  Favorable elements (P): proteins, fibers, fruits, vegetables, legumes and whole grains. |
| Carbohydrate Food Quality Score (CFQS)-5 | 1 point if fiber ≥ 10g/100g carb  1 point if free sugars < 10g/100g carb  1 point if Na < 600mg/100g dry weight  1 point if K > 300mg/100g dry weight  1 point if wholegrains ≥ 25g/100g dry weight | Favorable elements: fiber, potassium, wholegrains;  Unfavorable elements: free sugars, sodium. |

(DRI= dietary recommended intake; MRI= maximum recommended intake).

1. Nutritional quality across product groups

**Table S4**: Median value (IQR) of nutritional quality according to five numerical nutritional quality indices and whole grains content across grain-based food categories.

| **Indicator** | **All products**  **(n=356)** | **Flour, cereals, grains and crushed cereals**  **(n=62)** | **Rice**  **(n=28)** | **Breakfast cereals and muesli**  **(n=34)** | **Porridge and porridge powder**  **(n=23)** | **Soft bread and bread mixes**  **(n=35)** | **Rye bread, bread mixes based on rye**  **(n=8)** | **Hard bread and crusts**  **(n=41)** | **Pasta**  **(n=18)** | **Buns, soft cakes, pastry and other**  **(n=107)** |
| --- | --- | --- | --- | --- | --- | --- | --- | --- | --- | --- |
| CFQS-5 | 2.00  (1.00-  3.25) | 4.00  (3.00-5.00) | 2.00 (1.00-2.00) | 3.50  (1.00-  4.00) | 3.00  (2.00-3.00) | 2.00  (1.00-3.00) | 3.50  (2.50-4.00) | 3.00  (2.00-5.00) | 2.00  (2.00-2.75) | 1.00  (1.00-  2.00) |
| NRF11.3 | 43.23  (14.08–94.90) | 97.55  (52.49-159.51) | 16.72  (1.72-37.63) | 100.65  (48.40-167.01) | 19.57  (11.36-25.34) | 44.69  (34.19-57.58) | 65.70  (46.66-81.57) | 99.44  (61.26-130.78) | 26.10  (21.52-63.54) | 14.13  (-0.85-43.23) |
| NR-FI_carb_ | 3.69  (-7.42–13.29) | 17.98  (10.29-31.50) | 5.73  (0.82-8.88) | 12.08  (5.44-21.10) | 0.70  (-0.44-2.25) | 4.00  (2.04-5.05) | 7.33  (0.75-3.50) | 12.27  (3.67-23.23) | 5.13  (3.44-13.35) | -14.88  (-21.56 –  -7.43) |
| Nutri-Score | 4.00  (1–  15) | -3.00  (-5-  0) | 2.00  (1-3.25) | 7.50  (1-  13.25) | 3.00  (2-  3.50) | 4.00  (2-  5) | 1.50  (0.75-3.50) | 6.00  (1.00-12.00) | 0  (0-1.75) | 19.00  (14.00-23.00) |
| Nutri-Score_wholegrain_ | 5.50  (2-  15) | 0  (-3-  2) | 2.00  (1-  4) | 7.50  (4-  13.25) | 5.00  (3-  6.50) | 4.00  (2.50-  5) | 5.00  (3.00-5.25) | 6.00  (4.00-12.00) | 0.50  (0-2.00) | 19.00  (14.00-23.00) |
| Wholegrains (%) | 18.47  (0-  23) | 30.18  (0-  54.75) | 10.61  (0-  0) | 41.26  (0-  63.75) | 11.17  (0-  15) | 6.86  (0-  11) | 33.88  (12.75-53.25) | 46,93  (0-  100) | 5  (0-  0) | 2.08  (0-  0) |

1. Correlations among wholegrain content and nutritional quality

**Table S5**: Spearman correlations (ρ) for CFQS-5 and whole grains content (g per 100 g dry

weight).

| **Correlations** | | | | | |
| --- | --- | --- | --- | --- | --- |
|  | | | | CFQS | Wholegrain total (g) |
| Spearman's rho | | CFQS-5 | Correlation Coefficient | 1.000 | .645^**^ |
|  |  |  | Sig. (2-tailed) | . | <.001 |
|  |  |  | N | 356 | 356 |
|  |  | Wholegrain total (g) | Correlation Coefficient | .645^**^ | 1.000 |
|  |  |  | Sig. (2-tailed) | <.001 | . |
|  |  |  | N | 356 | 356 |
| **. Correlation is significant at the 0.01 level (2-tailed). | | | | | |

**Spearman's rho Correlations**

██ **Highly Positive:** *(None)*

██ **Positive:** *(CFQS-5 <---> Wholegrain total (g))*

██ **No Linear Correlation:** *(None)*

██ **Negative:** *(None)*

██ **Highly Negative:** *(None)*

**Figure S2 here.**

**Table S6**. Spearman correlations (ρ) for NR-FI_carb_ and whole grains content (g per 100 g dry weight).

| **Correlations** | | | | |
| --- | --- | --- | --- | --- |
|  | | | NR-FIcarb | Wholegrain total (g) |
| Spearman's rho | NR-FIcarb | Correlation Coefficient | 1.000 | .475^**^ |
|  |  | Sig. (2-tailed) | . | <.001 |
|  |  | N | 356 | 356 |
|  | Wholegrain total (g) | Correlation Coefficient | .475^**^ | 1.000 |
|  |  | Sig. (2-tailed) | <.001 | . |
|  |  | N | 356 | 356 |
| **. Correlation is significant at the 0.01 level (2-tailed).  **Spearman's rho Correlations**  ██ **Highly Positive:** *(None)*  ██ **Positive:** *(NR-FIcarb <---> Wholegrain total (g))*  ██ **No Linear Correlation:** *(None)*  ██ **Negative:** *(None)*  ██ **Highly Negative:** *(None)*  **Figure S3 here.** | | | | |

**Table S7**. Spearman correlations (ρ) for NRF11.3 and whole grains content (g per 100 g dry weight).

| **Correlations** | | | | |
| --- | --- | --- | --- | --- |
|  | | | NRF11.3 | Wholegrain total (g) |
| Spearman's rho | NRF11.3 | Correlation Coefficient | 1.000 | .452^**^ |
|  |  | Sig. (2-tailed) | . | <.001 |
|  |  | N | 356 | 356 |
|  | Wholegrain total (g) | Correlation Coefficient | .452^**^ | 1.000 |
|  |  | Sig. (2-tailed) | <.001 | . |
|  |  | N | 356 | 356 |
| **. Correlation is significant at the 0.01 level (2-tailed). | | | | |

**Spearman's rho Correlations**

██ **Highly Positive:** *(None)*

██ **Positive:** *(NRF11.3 <---> Wholegrain total (g))*

██ **No Linear Correlation:** *(None)*

██ **Negative:** *(None)*

██ **Highly Negative:** *(None)*

**Figure S4 here.**

**Table S8**. Spearman correlations (ρ) for Nutri-Score and whole grains content (g per 100 g dry weight).

| **Correlations** | | | | |
| --- | --- | --- | --- | --- |
|  | | | Score | Wholegrain total (g) |
| Spearman's rho | Nutri-Score | Correlation Coefficient | 1.000 | -.340^**^ |
|  |  | Sig. (2-tailed) | . | <.001 |
|  |  | N | 356 | 356 |
|  | Wholegrain total (g) | Correlation Coefficient | -.340^**^ | 1.000 |
|  |  | Sig. (2-tailed) | <.001 | . |
|  |  | N | 356 | 356 |
| **. Correlation is significant at the 0.01 level (2-tailed). | | | | |

**Spearman's rho Correlations**

██ **Highly Positive:** *(None)*

██ **Positive:** *(None)*

██ **No Linear Correlation:** *(None)*

██ **Negative:** *(Nutri-Score <---> Wholegrain total (g))*

██ **Highly Negative:** *(None)*

**Figure S5 here.**

**Table S9**. Spearman correlations (ρ) for Nutri-Score_wholegrain_ and whole grains content (g per 100 g dry weight).

| **Correlations** | | | | |
| --- | --- | --- | --- | --- |
|  | | | Score whole grain | Wholegrain total (g) |
| Spearman's rho | Nutri-Scorewholegrain | Correlation Coefficient | 1.000 | -.128^*^ |
|  |  | Sig. (2-tailed) | . | .016 |
|  |  | N | 356 | 356 |
|  | Wholegrain total (g) | Correlation Coefficient | -.128^*^ | 1.000 |
|  |  | Sig. (2-tailed) | .016 | . |
|  |  | N | 356 | 356 |
| *. Correlation is significant at the 0.05 level (2-tailed). | | | | |

**Spearman's rho Correlations**

██ **Highly Positive:** *(None)*

██ **Positive:** *(None)*

██ **No Linear Correlation:** *(None)*

██ **Negative:** *(Nutri-Scorewholegrain <---> Wholegrain total (g))*

██ **Highly Negative:** *(None)*

**Figure S6 here.**

1. Climate impact of grain-based foods

**Table S10**. Greenhouse gas emissions (median value (IQR)) per 100g product, unit of NRF11.3 and NR-FI_carb_ across grain-based food categories.

| **Indicator** | **All products**  **(n=356)** | **Flour, cereals, grains and crushed cereals**  **(n=62)** | **Rice**  **(n=28)** | **Breakfast cereals and muesli**  **(n=34)** | **Porridge and porridge powder**  **(n=23)** | **Soft bread and bread mixes**  **(n=35)** | **Rye bread, bread mixes based on rye**  **(n=8)** | **Hard bread and crusts**  **(n=41)** | **Pasta**  **(n=18)** | **Buns, soft cakes, pastry and other**  **(n=107)** |
| --- | --- | --- | --- | --- | --- | --- | --- | --- | --- | --- |
| kg CO2-eq/100 g product | 0.1  (0.05-0.16) | 0.06  (0.04-0.11) | 0.23  (0.11-0.31) | 0.16  (0.14-0.21) | 0.04  (0.01-0.10) | 0.05  (0.05-0.05) | 0.05  (0.05-0.05) | 0.05  (0.04-0.07) | 0.10  (0.04-0.1) | 0.14  (0.10-0.18) |
| kg CO2-eq/NRF11.3 | 0.0005  (0.0002-0.0008) | 0.0002  (0.0002-0.0004) | 0.0010  (0.0007-0.0014) | 0.0006  (0.0005-0.0008) | 0.0002  (0.00006-0.0006) | 0.0003  (0.0002-0.0003) | 0.0002  (0.0002-0.0003) | 0.0002  (0.0001-0.0003) | 0.0004  (0.0002-0.0005) | 0.0008  (0.0005-0.0011) |
| Kg CO2-eq/NR-FI_carb_ | 0.0021  (0.0009-0.0037) | 0.0008  (0.0006-0.0017) | 0.0035  (0.0023-0.0052) | 0.0028  (0.0022-0.0036) | 0.0008  (0.0002-0.0020) | 0.0009  (0.0009-0.0010) | 0.0009  (0.0008-0.0009) | 0.0007  (0.0006-0.0014) | 0.0016  (0.0008-0.0018) | 0.0041  (0.0028-0.0060) |

1. Water content of grain-based foods

**Table S11.** Water content (median value (IQR)) per 100g product across grain-based food categories.

| **Indicator** | **All products**  **(n=356)** | **Flour, cereals, grains and crushed cereals**  **(n=62)** | **Rice**  **(n=28)** | **Breakfast cereals and muesli**  **(n=34)** | **Porridge and porridge powder**  **(n=23)** | **Soft bread and bread mixes**  **(n=35)** | **Rye bread, bread mixes based on rye**  **(n=8)** | **Hard bread and crusts**  **(n=41)** | **Pasta**  **(n=18)** | **Buns, soft cakes, pastry and other**  **(n=107)** |
| --- | --- | --- | --- | --- | --- | --- | --- | --- | --- | --- |
| water (g/100 g) | 14.00  (7.00-37.13) | 13.25  (11.03-50.65) | 13.00  (10.68-70.08) | 4.35  (3.23-6.08) | 79.50  (76.85-82.25) | 35.20  (32.85-37.55) | 34.70  (33.28-36.78) | 5.50 (4.00-6.60) | 60.30  (10.53-66.10) | 21.10  (7.15-33.25) |

**References**

[1] Angelino D, Dinu M, Gandossi B, Pellegrini N, Martini D. Processing and Nutritional Quality of Breakfast Cereals Sold in Italy: Results from the Food Labelling of Italian Products (FLIP) Study. Nutrients 2023;15:2013. https://doi.org/10.3390/nu15082013.

[2] Bianchi M, Strid A, Winkvist A, Lindroos A-K, Sonesson U, Hallström E. Systematic Evaluation of Nutrition Indicators for Use within Food LCA Studies. Sustainability 2020;12:8992. https://doi.org/10.3390/su12218992.

[3] Byron C, Kissock KR, Barrett EM, Beck EJ. Aligning front-of-pack labelling with dietary guidelines: including whole grains in the health star rating. Eur J Nutr 2024;63:2025–33. https://doi.org/10.1007/s00394-024-03404-z.

[4] Comerford KB, Papanikolaou Y, Jones JM, Rodriguez J, Slavin J, Angadi S, et al. Toward an evidence-based definition and classification of carbohydrate food quality: An expert panel report. Nutrients 2021;13. https://doi.org/10.3390/nu13082667.

[5] Comerford KB, Drewnowski A, Papanikolaou Y, Jones JM, Slavin J, Angadi SS, et al. Application of a New Carbohydrate Food Quality Scoring System: An Expert Panel Report. Nutrients 2023;15. https://doi.org/10.3390/nu15051288.

[6] Curtain F, Grafenauer S. Health Star Rating in Grain Foods-Does It Adequately Differentiate Refined and Whole Grain Foods? Nutrients 2019;11. https://doi.org/10.3390/nu11020415.

[7] Darmon N, Sondey J, Azaïs-Braesco V, Maillot M. The SENS algorithm-a new nutrient profiling system for food labelling in Europe. Eur J Clin Nutr 2018;72:236–48. https://doi.org/10.1038/s41430-017-0017-6.

[8] Drewnowski A, Mckeown N, Kissock K, Beck E, Mejborn H, Vieux F, et al. Perspective: Why Whole Grains Should Be Incorporated into Nutrient-Profile Models to Better Capture Nutrient Density. Adv Nutr 2021;12:600–8. https://doi.org/10.1093/advances/nmaa172.

[9] Drewnowski A, Maillot M, Papanikolaou Y, Jones JM, Rodriguez J, Slavin J, et al. A New Carbohydrate Food Quality Scoring System to Reflect Dietary Guidelines: An Expert Panel Report. Nutrients 2022;14. https://doi.org/10.3390/nu14071485.

[10] Julia C, Kesse-Guyot E, Ducrot P, Peneau S, Touvier M, Mejean C, et al. Performance of a five category front-of-pack labelling system - the 5-colour nutrition label - to differentiate nutritional quality of breakfast cereals in France. BMC Public Health 2015;15:179/1. https://doi.org/10.1186/s12889-015-1522-y.

[11] Kårlund A, Kyttä V, Pellinen T, Tuomisto HL, Pajari A-M, Kolehmainen M, et al. Validating nutrient selection for product-group-specific nutrient indices for use as functional units in life cycle assessment of foods. Br J Nutr 2024;131:2049–57. https://doi.org/10.1017/S0007114524000709.

[12] Kissock KR, Vieux F, Mathias KC, Drewnowski A, Seal CJ, Masset G, et al. Aligning nutrient profiling with dietary guidelines: modifying the Nutri-Score algorithm to include whole grains. Eur J Nutr 2022;61:541–53. https://doi.org/10.1007/s00394-021-02718-6.

[13] Kyttä V, Kårlund A, Pellinen T, Tuomisto HL, Kolehmainen M, Pajari A-M, et al. Extending the product-group-specific approach in nutritional life cycle assessment. Int J Life Cycle Assess 2023b;30:93–109. https://doi.org/10.1007/s11367-023-02235-0.

[14] Øvrebø B, Brantsæter AL, Lund-Iversen K, Andersen LF, Paulsen MM, Abel MH. How does the updated Nutri-Score discriminate and classify the nutritional quality of foods in a Norwegian setting? Int J Behav Nutr Phys Act 2023;20:122. https://doi.org/10.1186/s12966-023-01525-y.

[15] Swedish Food Agency. Interpretations of Paragraphs and Food Groups. 2022.

[16] Robert M, Martin F, Xhonneux A, Mosser F, Favre E, Richonnet C. Nutritional Quality of Breakfast Cereals on the French, Belgian and Luxembourg Markets: Which Cereals for Children? Nutrients 2024;16. https://doi.org/10.3390/nu16162701.

[17] Sluik D, Streppel MT, van Lee L, Geelen A, Feskens EJM. Evaluation of a nutrient-rich food index score in the Netherlands. J Nutr Sci 2015;4:1–9. https://doi.org/10.1017/jns.2015.4.

[18] Vermote M, Bonnewyn S, Matthys C, Vandevijvere S. Nutritional Content, Labelling and Marketing of Breakfast Cereals on the Belgian Market and Their Reformulation in Anticipation of the Implementation of the Nutri-Score Front-Of-Pack Labelling System. Nutrients 2020;12. https://doi.org/10.3390/nu12040884.

[19] Wanselius J, Larsson C, Berg C, Öhrvik V, Lindroos AK, Lissner L. Consumption of foods with the Keyhole front-of-pack nutrition label: potential impact on energy and nutrient intakes of Swedish adolescents. Public Health Nutr 2022;25:3279–90. https://doi.org/10.1017/S1368980022002178.

[20] Swedish Food Agency. The Keyhole Design Manual. 2018.
